# Food Insecurity, Neighborhood Disorder, and Homelessness among People with Serious Mental Illness

**DOI:** 10.1007/s10597-025-01533-1

**Published:** 2025-11-07

**Authors:** Shiah Kleinman, Stacey Barrenger, Bailey Taylor, Khushbakht Shah, Tyler Chinsky, Alyssa Battaglia, Sanaiya Ahmed, Shivani Agarwal, Christina Abd, Natalie Bonfine

**Affiliations:** 1 Department of Psychiatry, Northeast Ohio Medical University, Rootstown, PO Box 95, 4209 SR 44, 44272 OH USA; 2 Department of Sociology and Criminology, Kent State University, Kent, OH 44242 USA

**Keywords:** Food insecurity, Neighborhood disorder, Homelessness, Social determinants, Serious mental illness

## Abstract

The health effects of social conditions such as income, education, and employment have been demonstrated to be persistent and wide-reaching. In this study, we examine the effect of social determinants of health, those conditions in which people live, among individuals with serious mental illnesses (SMI) who are actively engaged with mental health services. Using a sample of 203 clients at a community mental health clinic, this study (1) explores the prevalence of three social determinants of health: food insecurity, homelessness, and neighborhood disorder, and (2) assesses their associations with five outcomes of health, including mental health (overall mental health and life satisfaction) and physical health (overall physical health, number of chronic health conditions, and perceived daily limits). Our findings demonstrated that food insecurity and neighborhood disorder were prevalent within our sample of people with SMI (64% and 93% respectively), while homelessness was not (7%). Furthermore, a series of OLS regressions showed food insecurity and neighborhood disorder to be associated with poor mental and physical health, while homelessness was not significantly associated with any outcomes. These findings suggest that individuals with SMI who are actively engaged in treatment have increased risk of food insecurity and poor neighborhood conditions, which in turn may negatively impact their overall health. We suggest that mental health service providers be aware of the influence of social conditions on their patients and that clinics may be uniquely positioned to identify and intervene for individuals at risk of experiences that may be detrimental to their recovery.

**Supplementary Information:**

The online version contains supplementary material available at 10.1007/s10597-025-01533-1.

## Introduction

### The Roles and Risks of Social Determinants

Social determinants of health are social and structural conditions in which people live that can influence health outcomes, functioning, and quality of life (US Department of Health and Human Services, 2024). Social determinants of health may include socioeconomic conditions (e.g., income, job opportunities), educational attainment and opportunities, conditions that limit access to health care or reduce quality of care, or factors related to the environmental or social context (e.g., neighborhood conditions, safe and affordable housing, and access to healthy food options) (US Department of Health and Human Services, 2024). These social and structural factors put individuals at “risk of risks” (Link & Phelan, 1995, p. 80) and play a key role in determining health outcomes. Social determinants are linked to conditions of social and economic disadvantage, so it is important to explore the impact of these factors on health outcomes among those that may have disproportionate exposure to such risk, including people with serious mental illnesses (SMI), such as schizophrenia, bipolar disorder, or major depression. This population has been found to have increased risk of death or hospitalization due to poor health-related quality of life and increased comorbid health conditions (Young et al., 2024). Research exploring the prevalence of detrimental social conditions and their association with health outcomes may also present avenues for developing interventions at the structural and community levels to improve health. Additionally, understanding social determinants of health can impact the recovery experience of those with SMI (Compton et al., 2020).

### Exploring Social Determinants among People with SMI

Food insecurity refers to inconsistent or insufficient access to nutritious food through physical and/or economic barriers (Compton, 2014). Compton (2014) argued that while food insecurity may more obviously be considered a social determinant of physical health, it is an often-overlooked social determinant of mental health. In 2023, the United States Department of Agriculture (USDA) found that the national prevalence of low food security was 8.4% and very low food security was 5.1% (Rabbitt et al., 2024). Comparatively, in a sample of adults with SMI, Compton and Ku (2023) found the prevalence of low and very low food security to be 68.9% and 46.8% respectively. Jester and colleagues (2023) also noted that people with SMI experienced food insecurity 2.7 times more than the general population. This indicates that this population may be at particular risk for food insecurity which has been demonstrated to be associated with a number of negative mental health outcomes (Compton, 2014). For example, an analysis by Compton and colleagues (2020) found that both food insecurity and neighborhood disorder predicted a decrease in an index variable representing recovery among individuals with SMI.

Neighborhood disorder is conceptualized as characteristics of an individual’s neighborhood that indicate a lack of “peace, safety, and observance of the law” (Ross & Mirowsky, 1999, p. 413). Ross and Mirowsky (2001) found that individuals who live in such neighborhoods had worse physical health. People with SMI are more likely than the general population to live in neighborhoods characterized by structural disrepair, higher levels of drug and crime-related activities, and higher levels of social instability and isolation, which may ultimately impact health, functioning, participation, and community integration of people with SMI (Byrne et al., 2013; Jester et al., 2023). Importantly, their findings indicated that these neighborhood conditions were not solely the result of a higher concentration of individuals with SMI or that the areas were economically disadvantaged (Byrne et al., 2013). Ku and colleagues (2020) found that neighborhood disorder is associated with longer duration of untreated psychosis. Compton and colleagues (2020) found evidence that perceived neighborhood disorder—along with food insecurity and adverse childhood experiences—may be especially damaging to recovery for individuals with SMI. All together, these findings suggest that neighborhood disorder and food insecurity may be important influences on overall health; including in populations whose health may already be vulnerable.

Homelessness and housing instability—which covers a spectrum of housing-related situations from uncertainty of being able to maintain housing to being unhoused—has also been examined as a social determinant of mental health (Glasheen et al., 2019). Henwood and colleagues (2014) found that a housing first intervention had positive effects on domain-specific life satisfaction such as familial relationships and financial situation but not on overall life quality, suggesting a complex relationship between housing situations and subjective and objective experiences of quality of life. Notably, considerable research has focused on the prevalence of homelessness among people with SMI. As many as a quarter of those experiencing homelessness report SMI (Health Care for the Homeless, n.d.). Even among people with SMI who are connected to behavioral health services, it is estimated that 15% experience homelessness (Folsom et al., 2005).

### The Present Study

There is evidence to support conceptualizing food insecurity, experiences of homelessness, and neighborhood disorder as social determinants of health and mental health outcomes. In this study, we aimed to examine the prevalence and impact of social determinants among a sample of individuals with SMI who are actively engaged with community mental health services. In doing so, we sought to answer two broad research questions. First, we aimed to identify the prevalence of food insecurity, experiences of homelessness, and neighborhood disorder among our sample. Second, we sought to determine whether each individual social determinant of health is associated with physical and mental health outcomes for people with SMI who are engaged in treatment.

## Methods

### Sample

Our sample consists of 203 people with SMI who were recruited through a community mental health clinic in a large Midwestern city to participate in a survey. All participants were actively engaged in behavioral health services at a clinic that also offers integrated primary care and living independently in the surrounding community. A random sample was drawn from 1,500 active clients and informed about this study. Of those informed about the study, 223 people completed interviews (15%). Due to missing data on key measures, our final sample size for this analysis is 203. A comparative analysis (not shown here) found that, compared to the population of clients at the clinic, those in the sample were more likely to be female and received a higher proportion of services that were eligible to be covered by Medicaid. Exclusion criteria included individuals who did not speak English, were not at least 18 years old, were incarcerated, or were residents of a nursing home or psychiatric facility. Clients who were informed about the study were invited to contact the research team to schedule an in-person, quality of life survey that consisted of an interviewer-guided questionnaire. Each survey lasted about one-hour in total and participants received a $25-dollar gift card as compensation for their time. A chart review was also conducted to ascertain patients’ primary psychiatric diagnoses. Data were collected between 2013 and 2014.

### Measures

#### Food Insecurity

Food insecurity was measured using a modified Radimer-Cornell Hunger and Food Insecurity Scale (Kendall et al., 1995). Participants were asked to indicate whether the following statements were never true, often true, or sometimes true (range: 1–3): (1) “I worry whether my food will run out before I get money to buy more,” (2) “I eat the same thing for several days in a row because I only have a few different kinds of food on hand and don’t have money to buy more,” (3) “I ran out of the foods that I needed to put together a meal and I didn’t have money to get more food,” (4) “I can’t afford to eat properly,” (5) “I am often hungry, but I don’t eat because I can’t afford enough food,” and (6) “I eat less than I think I should because I don’t have enough money for food.” These items were combined to create a scale of food insecurity, with higher scores indicating greater food insecurity (α = 0.901, range 6–18).

#### Neighborhood Disorder

Neighborhood disorder was measured using Ross and Mirowsky’s Neighborhood Disorder Scale (Ross & Mirowksy, 1999), respondents were asked rate qualities of their neighborhoods on a four-point scale from “not at all” to “very much.” Questions asked to what degree one’s neighborhood (1) is noisy, (2) has vandalism, (3), has run-down houses or buildings, (4) has trash in the street, (5) has people hanging around in the streets, (6) has crime, (7) has alcohol and drug use, and (8) has heavy traffic. A scale assessing overall neighborhood disorder was created with higher scores indicating greater perceived neighborhood disorder (α = 0.857, range 8–32).

#### Homelessness

Homelessness was measured by creating a dichotomous measure, with “1” indicating that study respondents reported that they were homeless or living in a shelter at the time of the interview or, in the prior 12 months they had slept outside, slept in an empty building, slept in a public shelter, or slept in a church or mission (Lehman, 1988).

#### Mental Health Outcomes

Overall mental health was measured using a single-item measure from the National Comorbidity Survey where respondents were asked to rate their overall mental health on a five-point scale from poor to excellent (Kessler, 2008). Life satisfaction was measured using Diener and colleagues (1985) satisfaction with life scale and consists of five statements: (1), “in most ways my life is close to ideal,” (2) “the conditions of my life are excellent,” (3) “I am satisfied with my life,” (4) “so far, I have gotten the important things I want in life,” (5), “if I could live my life over, I would change almost nothing.” Response options ranged from strongly disagree to strongly agree. Alpha reliability for this scale is 0.788 (range 4–20).

#### Physical Health Outcomes

Like overall mental health, self-reported overall physical health was measured by asking respondents to rate their overall physical health on a five-point Likert scale, with responses ranging from “poor” to “excellent” (Kessler, 2008). To measure the number of chronic health conditions, we created a measure to count the number of physical health conditions they experience, such as chronic pain, heart disease, or cancer, among other chronic health conditions (range 0–15) (Kessler, 2008). Perceived daily limits were measured using one item from the Center of Disease Control’s (CDC) Healthy Days and Symptoms scale (Centers for Disease Control, n.d.) asking participants to indicate the number of days in the past month where their physical health kept them from doing daily activities (range 0–30 days).

### Analysis

Descriptive statistics, including frequencies, mean, standard deviation, were used to assess prevalence of social determinants of health (research question 1). A series of OLS regressions were run to determine significant associations among food insecurity, neighborhood disorder, and homelessness, and each of five mental and physical health outcome measures (research question 2). Data were analyzed using Stata/BE (version 18) (StataCorp, 2023).

### Compliance with Ethical Standards

Interviewers provided a complete description of the study, and all participants provided written informed consent for their involvement in the study. IRB approval was granted for this research study by the IRB at all participating academic institutions (Northeast Ohio Medical University and Kent State University). Approval was also granted by the research review committee at the participating community mental health clinic.

## Results

Full sample demographics can be found in Table 1. The age range was 25–72 (M = 49.52, SD = 9.605), most participants were white (58.13%) and the majority of the sample was female (61.58%). All participants were diagnosed with a SMI, with more than half of the sample having a principal diagnosis of schizophrenia (52.22%). About 21% of the sample had a diagnosis of major depressive disorder, 20% of the sample had a principal diagnosis of bipolar disorder, and 7% had some other diagnosis as their primary diagnosis for treatment. Most of the sample (83.3%) met eligibility requirements for Medicaid.

**Table 1:** Sample demographics (*N* = 203)

Variables	*N* (SD/%)
Age	49.52 (9.605)
Racial minority	85 (41.87)
Gender	125 (61.58)
Principal diagnosis	
Schizophrenia	106 (52.22)
Major depression	42 (20.7)
Bipolar disorder	40 (19.7)
Other principal diagnosis	15 (7.4)
Partnered	15 (7.39)
Children	114 (56.16)
Children under 18	27 (13.30)
Employed	27 (13.30)
Medicaid eligible	169 (83.3)

### Prevalence of Social Determinants

Our first research question explored the prevalence of each of the social determinants of health measures. About two-thirds of our sample (64%) reported some level of food insecurity. The average score on the food insecurity scale was 9.26 (SD = 3.448, range: 6 to 18). The three items with the greatest mean scores were related to worrying about running out of food before being able to buy more (M = 1.76, SD = 0.812, range = 1–3), running out of food and not having money to purchase more (M = 1.63, SD = 0.754, range = 1–3), and lacking the means to have nutritional variety (M = 1.57, SD = 0.764, range = 1–3).

Neighborhood disorder was also prevalent in this sample. The average score on the neighborhood disorder scale was 14.83 (SD = 5.429, range: 8 to 32). The three scale items most frequently reported were traffic in one’s neighborhood (M = 2.36, SD = 1.100), drug and alcohol use (M = 2.22, SD = 1.003), and crime (M = 1.98, SD = 0.962). Most of the sample (92.6%) indicated at least one undesirable neighborhood condition.

Experiencing homelessness was found to be relatively uncommon among this sample. Only 14 respondents (6.90%) had any indication of current or recent homelessness, such as indicating that they had slept in an empty building, public shelter, church or mission, or outside in the past twelve months. Otherwise, at the time of the interview, the majority of respondents (168, 83%) were living in their own home or apartment. Other respondents reported living with relatives or friends (19, 9%) and in supervised group living settings (14, 7%). Each of these categories of current living situation are indicative of a stable situation, preventing us from further exploring housing instability, separate from homelessness (Table 2).

**Table 2:** Descriptive statistics of measures (*N* = 203)

Variables	Mean (SD)/*N* (%)	Range	Alpha
***Independent variables***			
Neighborhood disorder scale	14.83 (5.429)	8–32	0.857
Food insecurity scale	9.26 (3.448)	6–18	0.901
Homelessness (1 = yes)	14 (6.90)	0–1	
***Dependent variables***			
Quality of life (satisfaction with life scale)	10.09 (2.260)	4–16	0.821
Overall mental health	2.64 (1.109)	1–5	
Overall physical health	2.48 (1.126)	1–5	
Number of chronic health conditions	3.71 (2.400)	0–10	
Perceived daily limits	7.50 (10.311)	0–30	

### Associations Between Social Determinants and Health Outcomes

Table 3 presents the results from a series of 15 different OLS regression analyses exploring the direct association of food insecurity, neighborhood disorder, and homelessness, each regressed on the five health outcomes measures: overall mental health, life satisfaction, overall physical health, the number of chronic health conditions reported, and limitations to activities of daily living (research question 2; 3 primary independent measures regressed on 5 different health outcome measures). Because our analysis involves multiple regression tests, we used a corrected p-value of 0.003, calculated using the Bonferroni method, to adjust the threshold of significance and reduce the potential bias that may occur from repeated testing. Across all regression equations, the Variance Inflation Factor did not exceed 1.25, and tolerance ranged from 0.815 to 0.967, above the threshold of 0.100, indicating that multicollinearity is not an issue in these findings. Breusch-Pagan tests for homoscedasticity were significant (p *≤* 0.001) for regression models with daily limitations regressed on food insecurity, neighborhood disorder, and homelessness, and the regression model with the number of chronic health conditions regressed on neighborhood disorder (*p* < 0.05), indicating heteroscedasticity for these equations. We conducted regression analyses with robust standard errors to correct for heteroscedasticity for these equations. Each OLS regression model controlled for the effect of participant age, gender, race, principal diagnosis, having a partner, parental status, and employment. The selection of control variables was based on exploratory correlational analyses between each demographic characteristic and the independent and dependent variables of interest (data available upon request). Demographic characteristics including education and Medicaid eligibility were excluded due to lack of association with key measures of interest. As our research question pertains to the direct associations among our key independent and dependent measures, that is, between our social determinants of health and outcome measures, the associations of the control measures are not included in our combined table (Table 3).

**Table 3:** Results of OLS regression models of mental and physical health on food Insecurity, neighborhood disorder and housing instability (*N* = 203)^a^

	Mental Health Outcomes				Physical Health Outcomes^b^					
	Overall mental health		Life satisfaction		Overall physical health		Number of chronic health conditions		Daily limitations	
	b(SE)	β	b(SE)	β	b(SE)	β	b(SE)	β	b(SE)	β
Food insecurity	−0.076***	− 0.240	−0.138**	− 0.217	−0.083***	− 0.264	0.160***	0.237	0.692	0.239
	(0.021)		(0.044)		(0.021)		(0.045)		(0.250)	
*Constant*	*3.403*		*10.677*		*3.329*		*− 0.114*		*6.231*	
*Adjusted R*^*2*^	*0.148*		*0.093*		*0.141*		*0.148*		*0.075*	
Neighborhood Disorder	−0.037	− 0.183	−0.105***	− 0.253	−0.037	− 0.178	0.097**	0.218	0.282	0.148
	(0.014)		(0.029)		(0.014)		(0.030)		(0.150)	
*Constant*	*3.201*		*10.968*		*3.113*		*0.014*		*9.245*	
Adjusted R^*2*^	*0.125*		*0.110*		*0.104*		*0.140*		*0.042*	
Homelessness	0.238	0.055	0.158	0.018	0.164	0.037	−0.287	− 0.030	−0.305	− 0.008
	(0.301)		(0.630)		(0.309)		(0.652)		(3.174)	
*Constant*	*2.501*		*9.046*		*2.334*		*1.791*		*14.378*	
*Adjusted R*^*2*^	*0.095*		*0.048*		*0.075*		*0.095*		*0.020*	

*** p *≤* 0.001, ** p *≤* 0.003. Using the Bonferroni method, we have calculated a corrected p-value of 0.003 to account for potential bias from repeated testing for the set of analyses

^a^Each sectioned row presents the unstandardized coefficient (standard error) and standardized coefficient (β) for the primary IV of different OLS regression models, each controlling for age, gender, race, principal diagnosis, having a partner, parental status, and employment status

^b^Regression with robust standard errors reported to correct for heteroscedasticity for models with daily limitations regressed on food insecurity, neighborhood disorder, and homelessness, and the model with the number of chronic health conditions regressed on neighborhood disorder

Food insecurity was found to be associated with four of five physical and mental health outcomes. Higher levels of food insecurity were negatively associated with overall mental health (b = −0.076, SE = 0.021, p *≤* 0.001, β = − 0.240, adjusted R^2^ = 0.148). Food insecurity was also negatively associated with life satisfaction when controlling for demographic variables (b = −0.138, SE = 0.044, *p* = 0.002, β = − 0.217). The adjusted R^2^ for this equation was 0.093, indicating that food insecurity accounted for about 9% of the variance in life satisfaction. Food insecurity was also found to be negatively associated with overall physical health (b = −0.083, SE = 0.021, p *≤* 0.001, β = − 0.264, adjusted R^2^ = 0.141, indicating about 14% of the variance in overall physical health is explained by food insecurity). Food insecurity was positively associated with the number of chronic health conditions (b = 0.160, SE = 0.045, *p* < 0.001, β = 0.237, adjusted R^2^ = 0.148, or about 15% of variance explained) when controlling for demographics. Food insecurity was not significantly associated with perceived daily limits when controlling for demographic characteristics.

Exploring the association of neighborhood disorder with two mental health outcomes, we found it was not significantly associated with overall mental health but found a significant, negative association with life satisfaction (b = −0.105, SE = 0.029, p *≤* 0.001, β = − 0.253), controlling for demographic factors. The adjusted R^2^ for this equation was 0.110, indicating about 11% of the variance in life satisfaction is due to neighborhood disorder. Exploring the association of neighborhood disorder with physical health outcomes, we found that neighborhood disorder was not significantly associated with overall physical health or daily limitations. Neighborhood disorder was significantly associated with an increased number of reported chronic health conditions (b = 0.097, SE = 0.030, p *≤* 0.003, β = 0.218 adjusted R^2^ = 0.140), controlling for demographic factors.

The association between homelessness and overall mental health and life satisfaction were not statistically significant (b = 0.238, SE = 0.301, *p* = 0.443, β = 0.055, adjusted R^2^ = 0.095 and b = 0.158, SE = 0.630, *p* = 0.815, β = 0.018, adjusted R^2^ = 0.048 respectively), controlling for demographic variables. Similarly, homelessness was not significantly associated with overall physical health (b = 0.164, SE = 0.309, *p* = 0.502, β = 0.037, adjusted R^2^ = 0.075), number of chronic health conditions (b = −0.287, SE = 0.652, *p* = 0.495, β = − 0.030 adjusted R^2^ = 0.095), and daily limitations (b = −0.305, SE = 3.174, *p* = 0.785, β = − 0.008 adjusted R^2^ = 0.020).

## Discussion

Food insecurity, homelessness, and neighborhood disorder have all been identified as social determinants of health (Compton, 2014; Compton et al., 2020; Padgett, 2020). However, gaps remain in identifying these social determinants among specific populations and in understanding how they may impact different dimensions of health. We sought to investigate the prevalence of these social conditions and assess whether these experiences were associated with both mental and physical health outcomes among adults with SMI engaged in treatment; a population that may be at higher risk of experiencing such adverse social conditions (Byrne et al., 2013; Compton et al., 2020).

Two of the three social determinants, specifically food insecurity and neighborhood disorder, were found to be common among our sample of people with SMI. Only about one third (36%) of our sample was food secure, with 64% of our sample responding affirmatively to at least one indicator of food insecurity. These findings support prior research indicating that individuals with SMI may be at higher risk of food insecurity (Compton & Ku, 2023). In 2023, 14% of all U.S. households in the general population had some indication of food insecurity (Rabbitt et al., 2024). Thus, it is evident that people with SMI may disproportionately face increased obstacles in regularly securing access to food.

Neighborhood disorder was also prevalent, as 93% of our sample reported some level of disorder in their neighborhood. These findings are consistent with prior research that has found higher rates of disorder in the neighborhoods where those with SMI are likely to live (Byrne et al., 2013; Compton et al., 2020), and an impact of perceived neighborhood disorder on the duration of untreated psychosis (Ku et al., 2020). Given the prevalence of perceived neighborhood disorder within this sample, future research should consider neighborhood conditions as possible factors that affect behavioral health care access and utilization. In contrast to this, homelessness was found to be less common, with only 7% of our sample reporting any indication of homelessness at the time of the interview or within the prior 12 months. Our sample represents people with SMI who were connected to community-based mental health services and living in non-institutionalized settings. Recent research by Yuan and Manuel (2024) found that service engagement is inversely associated with housing instability. As such, this sample may be relatively stable compared to people with SMI who are not connected to services or those who may be living in institutionalized settings. Future research should explore the prevalence of homelessness, especially among people with SMI who are connected to behavioral health services, as well as housing insecurity, a broader concept which may also capture a lack of stable occupancy or lack of safe or affordable housing (Murdoch et al., 2022).

Our findings exploring direct associations among food insecurity and neighborhood disorder and health outcomes suggest a persistent, significant association between these social conditions and poor physical and mental health, even among a sample of people with SMI who are engaged with behavioral health services. Specifically, we found that food insecurity is associated with worse mental health, reduced life satisfaction, poorer overall physical health and a higher number of chronic health conditions. Neighborhood disorder is associated with reduced life satisfaction and an increase in the number of chronic health conditions. These results are concerning given research that underscores an increased risk of death or hospitalization among people with SMI who have poor health or comorbid health conditions (Young et al., 2024). Homelessness was not significantly associated with any health outcome measures within this sample.

Food insecurity is prevalent and associated with poorer physical and mental health among a population of people with SMI who are engaged in services. Mental health service providers and primary care providers who treat people with SMI should assess levels of food insecurity among clients and work to connect those in need with available resources, including Medicaid and programs that support access to food such as the Supplemental Nutrition Assistance Program (SNAP), the Supplemental Nutrition Program for Women, Infants, and Children (WIC), local food pantries, or other community-based resources. Other approaches to addressing food insecurity include improving access to nutritious food and produce within the local community. Research has shown promising results associated with locating food pantries within clinics and hospitals (Greenthal et al., 2019; Reinoso et al., 2022). In particular, Greenthal and colleagues (2019) found that clients of a hospital reported greater confidence in the nutritional quality and safety of the food they received as well as less stigma when utilizing food pantry services. Other models, such as providing food-insecure individuals with redeemable vouchers at local farmers’ markets have found some success in increasing fruit and vegetable intake (Dailey et al., 2015). Such programs should be targeted towards people engaged in the community mental health system.

Poor neighborhood conditions were also significantly associated with worse health outcomes, specifically a higher number of chronic health conditions and reduced life satisfaction, indicating an opportunity to improve population health outcomes by addressing neighborhood conditions. This may involve addressing the social environment by encouraging community-building activities. In conducting interviews with people with psychiatric disabilities, Townley (2015) found that community mental health providers may be uniquely positioned to serve as a bridge between clients and the broader community to promote community integration due to their ability to build relationships with clients. Townley’s (2015) participants described receiving help with transportation from clinic staff and having friendly interactions with staff members in nonclinical settings. Programs like Project Connect may serve as useful models (Bromage et al., 2017). In Project Connect, staff members serve as facilitators of connections for clients, they identify an individual’s interests in an introductory meeting and assist the individual in locating local groups where these interests can be pursued. Bromage and colleagues’ implementation of Project Connect found some success in connecting clients with volunteer opportunities, hobby groups, and other groups and associations that allowed individuals to build relationships on equal footing with community members. Additionally, community leaders also have a role in increasing community engagement through creating a welcoming atmosphere and eliminating barriers like sharing information about opportunities for involvement (Bromage et al., 2018). Creating welcoming places (Snethen et al., 2021) in the community for people with SMI can increase a sense of belonging contributing to a favorable neighborhood social climate which is more important to psychological wellbeing than neighborhood experiences like safety and satisfaction (Kloos & Townley, 2011).

Neighborhood conditions may also be improved by addressing the physical environment. For example, community groups that facilitate the construction and maintenance of community gardens present an opportunity to build or strengthen social networks through the sharing of materials and knowledge, which can contribute to greater social support (Okvat & Zautra, 2011). An evaluative study of six community gardens in North Carolina conducted by Sadeghzadeh and colleagues (2022) found that garden participants identified a range of social and community benefits including passing down cultural knowledge through food, developing relationships in their immediate and broader communities, and holding community events. Such community efforts may address issues of food scarcity and insecurity in neighborhoods that are considered food deserts while also improving neighborhood conditions. Other interventions to improve neighborhood conditions may focus on revitalizing the built environment and improving safety within disadvantaged neighborhoods. Strategies to improve the built environment may include engaging volunteers to repair vacant homes, assist community members through housing counseling, providing emergency repair funds, and increased efforts to keep neighborhoods clean (YNDC, 2024). Strategies for improving public safety may include canvassing and building relationships with community members who were eligible for free security lights and doorbell cameras if they agreed to share any incident footage with the local police department (YNDC, 2024), or by in engaging in community policing, an approach to policing that builds relationships between local law enforcement officers and community members (Gill et al., 2014; Office of Community Oriented Policing Services, 2014). Strategies of community policing include holding regularly scheduled, local community meetings where police and community members can meet to exchange ideas and build relationships, or to provide training and education for community members about their role in community policing and crime prevention (Skogan, 1996, 2019).

### Limitations and Future Directions

While there are notable strengths to this study, including our approach to draw a sample from a large pool of people with SMI who are engaged in the community mental health treatment system, there are some limitations to this study. Our cross-sectional data were collected in 2013 and 2014 and offer a snapshot of social factors affecting health outcomes at that time. Recent social, environmental and living conditions, notably the global pandemic, may affect current associations among these concepts. Further, our analyses are cross-sectional and do not allow for causal determinations of the associations discussed. While we asked about an array of social factors affecting the lives of our participants, our measure of homelessness may be underestimating more tenuous or transient housing situations that could signal housing instability. Future research should examine a more expanded conceptualization of housing instability among people with SMI. Future research may also expand upon the conceptualization of neighborhood disorder by assessing more objective measures of environment conditions see Ku et al., 2021). Additionally, because of our focus on the individual associations between social determinants and health outcomes, our inclusion of variables such as gender and race was only for the purpose of statistical controls. Future research should examine the intersectional impacts of various social determinants and social identities and roles as prior research suggests that certain populations, such as Black and Latinx individuals, may be at heightened risk of social determinants which may, in turn, contribute to mental health outcomes (Anglin et al., 2021; Anglin, 2023). Finally, our focus for this analysis was determining direct associations between social conditions and health outcomes, and future research should also explore mediating processes that may identify the mechanisms and processes for how social conditions elicit physical and mental health impacts.

## Conclusion

It is well known and documented that social and structural conditions shape population health and wellbeing. Exploring these patterns is essential as people with SMI have an elevated risk of exposure to social conditions that negatively impact overall health. Our findings suggest that people with SMI have high levels of food insecurity and poor or unsafe neighborhood conditions, which are associated with poor physical and mental health outcomes. Understanding the social and living conditions of people with SMI is an important step towards building interventions and programming that can interrupt their harmful effect on health outcomes. Healthcare providers, and especially the community mental health system, have an opportunity to expand its approach to meet the social and mental health needs of clients by understanding the prevalence of social determinants of health among their clients, especially food insecurity and poor neighborhood conditions, and by implementing programming and policy to reduce the impact of health-related social needs.

## Supplementary Information

Below is the link to the electronic supplementary material.

ESM1 (DOCX 483 KB)

## References

[CR1] Anglin, D. M. (2023). Racism and social determinants of psychosis. *Annual Review of Clinical Psychology,**19*, 277–302. 10.1146/annurev-clinpsy-080921-074730 36888999 10.1146/annurev-clinpsy-080921-074730

[CR2] Anglin, D. M., Ereshefsky, S., Klaunig, M. J., Bridgwater, M. A., Niendam, T. A., Ellman, L. M., DeVylder, J., Thayer, G., Bolden, K., Musket, C. W., Grattan, R. E., Lincoln, S. H., Schiffman, J., Lipner, E., Bachman, P., Corcoran, C. M., Mota, N. B., & Van Der Ven, E. (2021). From womb to neighborhood: A racial analysis of social determinants of psychosis in the United States. *American Journal of Psychiatry,**178*(7), 599–610. 10.1176/appi.ajp.2020.20071091 33934608 10.1176/appi.ajp.2020.20071091 PMC8655820

[CR3] Bromage, B., Kriegel, L., Williamson, B., Maclean, K., & Rowe, M. (2017). Project connect: A community intervention for individuals with mental illness. *American Journal of Psychiatric Rehabilitation,**20*(3), 218–233. 10.1080/15487768.2017.1338038

[CR4] Bromage, B., Barrenger, S. L., Clayton, A., Rowe, M., Williamson, B., & Kriegel, L. S. (2018). Facilitating community connections among people with mental illnesses: Perspectives from grassroots community leaders. *Journal of Community Psychology,**47*, 663–678. 10.1002/jcop.22146 30500066 10.1002/jcop.22146

[CR5] Byrne, T., Prvu Bettger, J., Brusilovskiy, E., Wong, Y. L. I., Metraux, S., & Salzer, M. S. (2013). Comparing neighborhoods of adults with serious mental illness and of the general population: Research implications. *Psychiatric Services,**64*(8), 782–788. 10.1176/appi.ps.201200365 23677444 10.1176/appi.ps.201200365

[CR6] Centers for Disease Control and Prevention. (n.d.). *Measuring healthy days: Population assessment of health-related quality of life*. Retrieved June 30 (2025). from https://archive.cdc.gov/www_cdc_gov/hrqol/hrqol14_measure.htm

[CR7] Compton, M. T. (2014). Food insecurity as a social determinant of mental health. *Psychiatry Annals,**44*(1), 46–51. 10.3928/00485713-20140108-08

[CR8] Compton, M. T., & Ku, B. S. (2023). Prevalence of food insecurity and living in a food desert among individuals with serious mental illnesses in public mental health clinics. *Community Mental Health Journal,**59*(2), 357–362. 10.1007/s10597-022-01013-w 35963919 10.1007/s10597-022-01013-w PMC10209833

[CR9] Compton, M. T., Bakeman, R., Capulong, L., Pauselli, L., Alolayan, Y., Crisafio, A., King, K., Reed, T., Broussard, B., & Shim, R. (2020). Associations between two domains of social adversity and recovery among persons with severe mental illness being treated in community mental health centers. *Community Mental Health Journal*, *56*, 22–31. 10.1007/s10597-019-00462-0 31552538 10.1007/s10597-019-00462-0

[CR10] Dailey, A. B., Hess, A., Horton, C., Constantian, E., Monani, S., Wargo, B., Davidson, K., & Gaskin, K. (2015). Healthy options: A community-based program to address food insecurity. *Journal of Prevention & Intervention in the Community*, *43*(2), 83–94. 10.1080/10852352.2015.973248 25898216 10.1080/10852352.2015.973248

[CR11] U.S. Department of Health and Human Services (2024). *Healthy people 2030.* Office of Disease Prevention and Health Promotion. Retrieved December 23, 2024, from https://odphp.health.gov/healthypeople/objectives-and-data/social-determinants-health

[CR12] Diener, E., Emmons, R. A., Larsen, R. J., & Griffin, S. (1985). The satisfaction with life scale. *Journal Of Personality Assessment,**49*(1), 71–75. 10.1207/s15327752jpa4901_13 16367493 10.1207/s15327752jpa4901_13

[CR13] Folsom, D. P., Hawthorne, W., Lindamer, L., Gilmer, T., Bailey, A., Golshan, S., Garcia, P., Unutzer, J., Hough, R., & Jeste, D. V. (2005). Prevalence and risk factors for homelessness and utilization of mental health services among 10,340 patients with serious mental illness in a large public mental health system. *American Journal of Psychiatry,**162*(2), 370–376. 10.1176/appi.ajp.162.2.370 15677603 10.1176/appi.ajp.162.2.370

[CR14] Gill, C., Weisburd, D., Telep, C. W., Vitter, Z., & Bennett, T. (2014). Community-oriented policing to reduce crime, disorder and fear and increase satisfaction and legitimacy among citizens: A systematic review. *Journal of Experimental Criminology,**10*, 399–428. 10.1007/s11292-014-9210-y

[CR15] Glasheen, C., Forman-Hoffman, V. L., Hedden, S., Ridenour, T. A., Wang, J., & Porter, J. D. (2019). Residential transience among adults: Prevalence, characteristics, and association with mental illness and mental health service use. *Community Mental Health Journal,**55*, 784–797. 10.1007/s10597-019-00385-w 30859359 10.1007/s10597-019-00385-w

[CR16] Greenthal, E., Jia, J., Poblacion, A., & James, T. (2019). Patient experiences and provider perspectives on a hospital-based food pantry: A mixed methods evaluation study. *Public Health Nutrition,**22*(17), 3261–3269. 10.1017/S1368980019002040 31486351 10.1017/S1368980019002040 PMC10260686

[CR17] Health Care for the Homeless. (n.d.). *Homelessness makes you sick.* Retrieved October 15 (2024). from https://www.hchmd.org/homelessness-makes-you-sick

[CR18] Henwood, B. F., Matejkowski, J., Stefancic, A., & Lukens, J. M. (2014). Quality of life after *housing first* for adults with serious mental illness who have experienced chronic homelessness. *Psychiatry Research,**1*, 549–555. 10.1016/j.psychres.2014.07.072 10.1016/j.psychres.2014.07.072 25129560

[CR19] Jester, D. J., Thomas, M. L., Sturm, E. T., Harvey, P. D., Keshavan, M., Davis, B. J., Saxena, S., Tampi, R., Leutwyler, H., Compton, M. T., Palmer, B. W., & Jeste, D. V. (2023). Review of major social determinants of health in schizophrenia-spectrum psychotic disorders: I. Clinical outcomes. *Schizophrenia Bulletin,**49*(4), 837–850. 37022779 10.1093/schbul/sbad023 PMC10318890

[CR20] Kendall, A., Olson, C. M., & Frongillo, E. A. (1995). Validation of the Radimer/Cornell measures of hunger and food insecurity. *The Journal of Nutrition,**125*(11), 2793–2801. 10.1093/jn/125.11.2793 7472659 10.1093/jn/125.11.2793

[CR21] Kessler, R. C. (2008). National comorbidity survey: Baseline (NCS-1), 1990–1992. Inter-university Consortium for Political and Social Research [distributor], 2008-09-12. 10.3886/ICPSR06693.v6

[CR22] Kloos, B., & Townley, G. (2011). Investigating the relationship between neighborhood experiences and psychiatric distress for individuals with serious mental illness. *Administration and Policy in Mental Health and Mental Health Services Research*, *38*(2), 105–111. 10.1007/s10488-010-0307-y 20680675 10.1007/s10488-010-0307-y

[CR23] Ku, B. S., Pauselli, L., Manseau, M., & Compton, M. T. (2020). Neighborhood-level predictors of age at onset and duration of untreated psychosis in first-episode psychotic disorders. *Schizophrenia Research,**218*, 247–254. 10.1016/j.schres.2019.12.036 31948900 10.1016/j.schres.2019.12.036 PMC7299734

[CR24] Ku, B. S., Compton, M. T., Walker, E. F., & Druss, B. G. (2021). Social fragmentation and schizophrenia: A systematic review. *Journal of Clinical Psychiatry*, *83*(1). 10.4088/JCP.21r13941 10.4088/JCP.21r13941 PMC13103956 34875149

[CR25] Lehman, A. F. (1988). A quality of life interview for the chronically mentally ill. *Evaluation and Program Planning,**11*(1), 51–62.

[CR26] Link, B. G., & Phelan, J. (1995). Social conditions as fundamental causes of disease. *Journal of Health and Social Behavior*, (Extra Issue), 80–94. 7560851

[CR27] Murdoch, J., Meghna, B., Dennis, O., Moumen, F., & Streiff, S. (2022). *Measuring housing insecurity: Index development using American housing survey data*. U.S. Department of Housing and Urban Development.

[CR28] Office of Community Oriented Policing Services. (2014). *Community-oriented policing defined*. U.S. Department of Justice, Office of Community Oriented Policing Services.

[CR29] Okvat, H. A., & Zautra, A. J. (2011). Community gardening: A parsimonious path to individual, community, and environmental resilience. *American Journal of Community Psychology,**47*(3), 374–387. 10.1007/s10464-010-9404-z 21222153 10.1007/s10464-010-9404-z

[CR30] Padgett, D. K. (2020). Homelessness, housing instability and mental health: Making the connections. *BJPsych Bulletin,**44*(5), 197–201. 10.1192/bjb.2020.49 32538335 10.1192/bjb.2020.49 PMC7525583

[CR31] Rabbitt, R. P., Hales, L. J., & Reed-Jones, M. (2024). *Food security in the U.S.: Key statistics and graphs. USDA Economic Research Service*. https://www.ers.usda.gov/topics/food-nutrition-assistance/food-security-in-the-u-s/key-statistics-graphics/

[CR32] Reinoso, D., Haut, D., Claffey, S., Hahn Keiner, K., Chavez, A., Nace, N., & Carter, A. (2022). Addressing food insecurity: Lessons learned from co-locating a food pantry with a federally qualified health center. *International Journal of Integrated Care*, *22*(3), 1–11. 10.5334/ijic.6430 10.5334/ijic.6430 PMC9524299 36248069

[CR33] Ross, C. E., & Mirowsky, J. (1999). Disorder and decay: The concept and measurement of perceived neighborhood disorder. *Urban Affairs Review,**34*(3), 412–432.

[CR34] Ross, C. E., & Mirowsky, J. (2001). Neighborhood disadvantage, disorder, and health. *Journal of Health and Social Behavior,**42*(3), 258–276. 11668773

[CR35] Sadeghzadeh, C., Sheppard, B., de Groot, J., & De Marco, M. (2022). Evaluating the benefits of a SNAP-Ed-funded community garden intervention using ripple effect mapping. *Health Education & Behavior*, *49*(1), 141–149. 10.1177/10901981211058075 34963366 10.1177/10901981211058075

[CR36] Skogan, W. G. (2019). Advocate: Community policing. Pp. 27–44 in *Police Innovation: Contrasting* Perspectives 2^nd^ Edition. Edited by David Weisburg and Anthony Braga. Cambridge University Press.

[CR37] Skogan, W. G. (1996). The community’s role in community policing. *National Institute of Justice Journal,**231*, 31–34.

[CR38] Snethen, G., Jeffries, V., Thomas, E., & Salzer, M. (2021). Welcoming places: Perspectives of individuals with mental illness. *American Journal of Orthopsychiatry*, *91*(1), 76–85. 10.1037/ort000519 33411553 10.1037/ort0000519

[CR39] StataCorp. (2023). *Stata statistical software: Release 18*. StataCorp LLC.

[CR40] Townley, G. (2015). It helps you not feel so bad—feel like you again: The importance of community for individuals with psychiatric disabilities. *Journal of Psychosocial Rehabilitation and Mental Health,**2*(2), 113–124. 10.1007/s40737-015-0036-3

[CR41] YNDC (2024). Performance report: January-March 2024. https://www.yndc.org/sites/default/files/Q1_PerformanceReport_2024W__0.pdf

[CR42] Young, A. S., Skela, J., & Siddarth, P. (2024). The characteristics of people with serious mental illness who are at high risk for hospitalization or death. *Community Mental Health Journal,**60*, 1243–1246. 38653869 10.1007/s10597-024-01281-8

[CR43] Yuan, Y., & Manuel, J. (2024). Factors associated with housing stability among individuals with co-occurring serious mental illness and substance use disorders receiving assertive community treatment services. *Community Mental Health Journal Doi*10.1007/s10597-024-01443-8 10.1007/s10597-024-01443-8 PMC12228652 39739210

