# Supplementary material for: Food Insecurity, Neighborhood Disorder, and Homelessness among People with Serious Mental Illness
Source: Community Ment Health J. 2025 Nov 7;62(2):385–94. doi: 10.1007/s10597-025-01533-1 (PMC12852297; doi:10.1007/s10597-025-01533-1)
Supplement: Supplementary file 1 — (DOCX 483 KB) [file 10597_2025_1533_MOESM1_ESM.docx]

The following tables and figures accompany the second re-submission for the paper entitled “Food Insecurity, Neighborhood Disorder, and Homelessness among People with Serious Mental Illnesses Currently Engaged with Community Mental Health Services” (#COMH-S-25-00136).

**Supplemental Table 1. Skewness and kurtosis values of independent and dependent measures^a^**

**^a^** Homelessness, a dichotomous measure, is not included in this analysis.

| **Supplemental Table 2. Results of Multivariate OLS Regression Model of Mental and Physical Health on Food Insecurity (N=203)** | | | | | |
| --- | --- | --- | --- | --- | --- |
| Mental Health Outcomes | | | Physical Health Outcomes | | |
|  | Overall mental health | Life satisfaction | Overall physical health | Number of chronic health conditions | Daily limitations |
|  | b  (SE) | b  (SE) | b  (SE) | b  (SE) | b  (SE) |
| Food insecurity | -0.075***  (0.210) | -0.138**  (0.044) | -0.083***  (0.021) | 0.158***  (0.045) | 0.687***  (0.202) |
| *Constant* | 3.428  (0.509) | 10.716  (1.070) | 3.224  (0.517) | 0.274  (1.088) | 7.455  (4.900) |
| *R^2^* | 0.182 | 0.129 | 0.181 | 0.201 | 0.122 |

*** p<.001, ** p<.01. Each model included controls for age, gender, race, principal diagnosis, having a partner, parental status, and employment status.

| **Supplemental Table 3. Results of Multivariate OLS Regression Model of Mental and Physical Health on Neighborhood Disorder (N=203)** | | | | | |
| --- | --- | --- | --- | --- | --- |
| Mental Health Outcomes | | | Physical Health Outcomes | | |
|  | Overall mental health | Life satisfaction | Overall physical health | Number of chronic health conditions | Daily limitations |
|  | b  (SE) | b  (SE) | b  (SE) | b  (SE) | b  (SE) |
| Neighborhood Disorder | -0.038  (0.014) | -0.108***  (0.029) | -0.035  (0.014) | 0.090**  (0.030) | 0.260  (0.136) |
| *Constant* | 3.268  (0.522) | 11.118  (1.073) | 2.907  (0.536) | 0.450  (1.112) | 10.660  (5.064) |
| *R^2^* | 0.161 | 0.147 | 0.143 | 0.188 | 0.087 |

*** p<.001, ** p<.01. Each model included controls for age, gender, race, principal diagnosis, having a partner, parental status, and employment status.

| **Supplemental Table 4. Results of Multivariate OLS Regression Model of Mental and Physical Health on Homelessness (N=203)** | | | | | |
| --- | --- | --- | --- | --- | --- |
| Mental Health Outcomes | | | Physical Health Outcomes | | |
|  | Overall mental health | Life satisfaction | Overall physical health | Number of chronic health conditions | Daily limitations |
|  | b  (SE) | b  (SE) | b  (SE) | b  (SE) | b  (SE) |
| Homelessness | 0.233  (0.304) | 0.149  (0.635) | 0.209  (0.311) | -0.445  (0.649) | -0.780  (2.921) |
| *Constant* | 2.513  (0.460) | 9.071  (0.961) | 2.217  (0.470) | 2.201  (0.983) | 16.552  (4.422) |
| *R^2^* | 0.131 | 0.086 | 0.119 | 0.152 | 0.070 |

*** p<.001, ** p<.01. Each model included controls for age, gender, race, principal diagnosis, having a partner, parental status, and employment status.

**Supplemental Table 5. Changes in adjusted R^2^ to reflect effect of key independent variables over control-only models**

|  | Model 1 adjusted R^2^ (controls only) | Model 2 adjusted R^2^ (adding in key IV) | Δ adjusted R^2^ |
| --- | --- | --- | --- |
| Food insecurity 🡪 overall mental health | .097 | .148 | +.051 |
| Food insecurity 🡪 life satisfaction | .052 | .093 | +.041 |
| Food insecurity 🡪 overall physical health | .078 | .141 | +.063 |
| Food insecurity 🡪 # of chronic health conditions | .098 | .148 | +.050 |
| Food insecurity 🡪 daily limitations | .025 | .075 | +.050 |
| Neighborhood disorder 🡪 overall mental health | .097 | .125 | +.028 |
| Neighborhood disorder 🡪 life satisfaction | .052 | .110 | +.058 |
| Neighborhood disorder 🡪 overall physical health | .078 | .104 | +.026 |
| Neighborhood disorder 🡪 # of chronic health conditions | .098 | .140 | +.042 |
| Neighborhood disorder 🡪 daily limitations | .025 | .042 | +.017 |
| Homelessness 🡪 overall mental health | .097 | .095 | -.002 |
| Homelessness 🡪 life satisfaction | .052 | .048 | -.004 |
| Homelessness 🡪 overall physical health | .078 | .075 | -.003 |
| Homelessness 🡪 # of chronic health conditions | .098 | .095 | -.003 |
| Homelessness 🡪 daily limitations | .025 | .020 | -.005 |

**Scatterplots with fit lines**

| **Food insecurity and overall mental health**  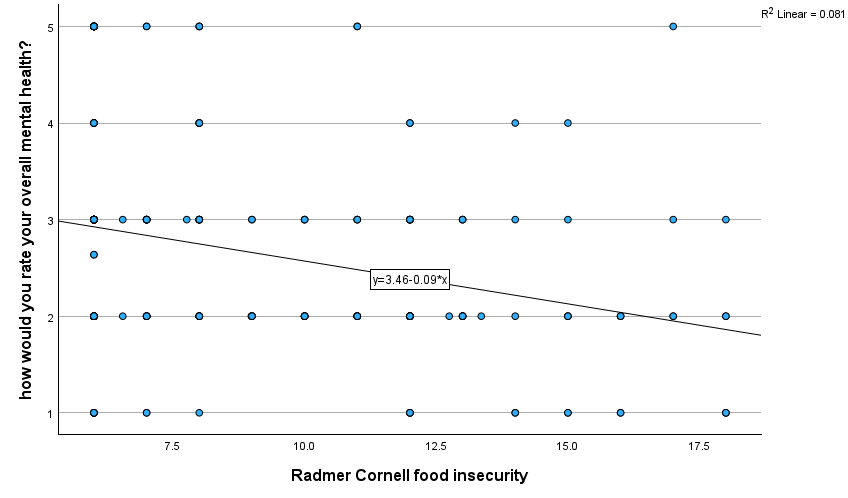 |
| --- |
| **Food insecurity and life satisfaction**  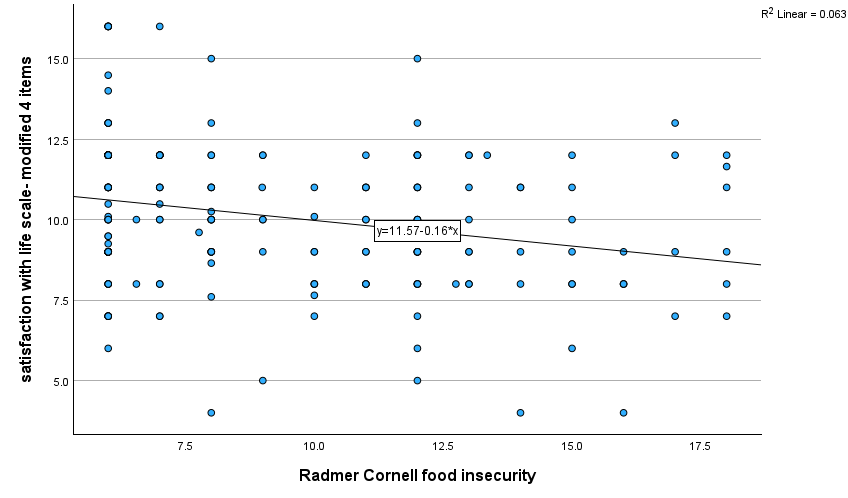 |
| **Food insecurity and overall physical health**  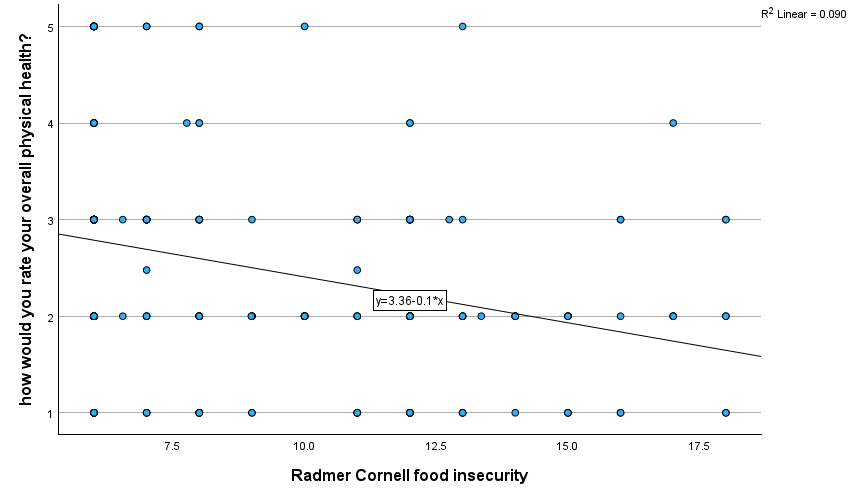 |
| **Food insecurity and # of chronic health conditions**  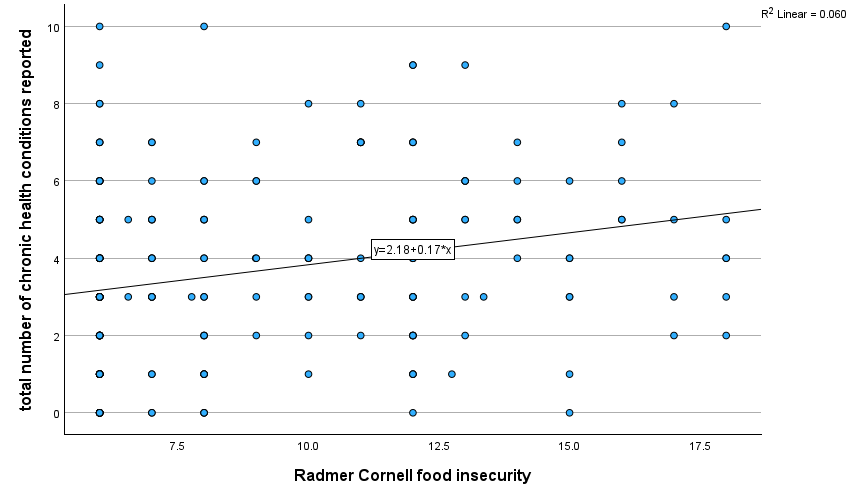 |
| **Food insecurity and daily limitations** |
| 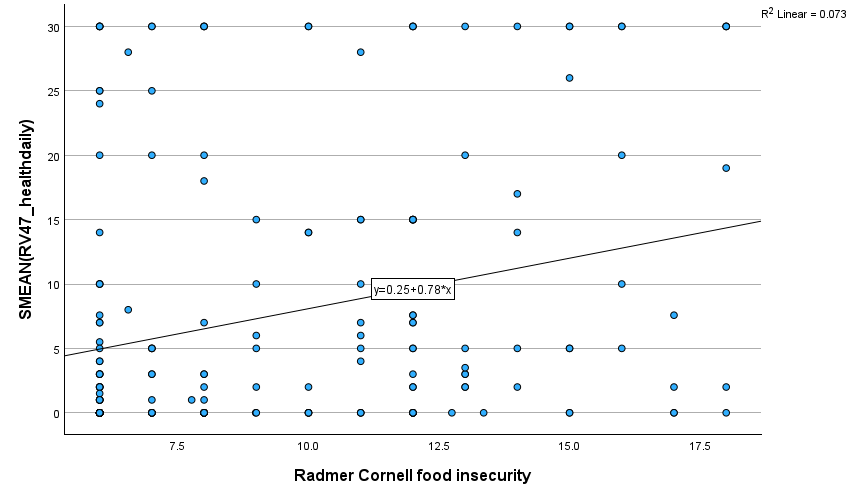  **Neighborhood disorder and overall mental health**  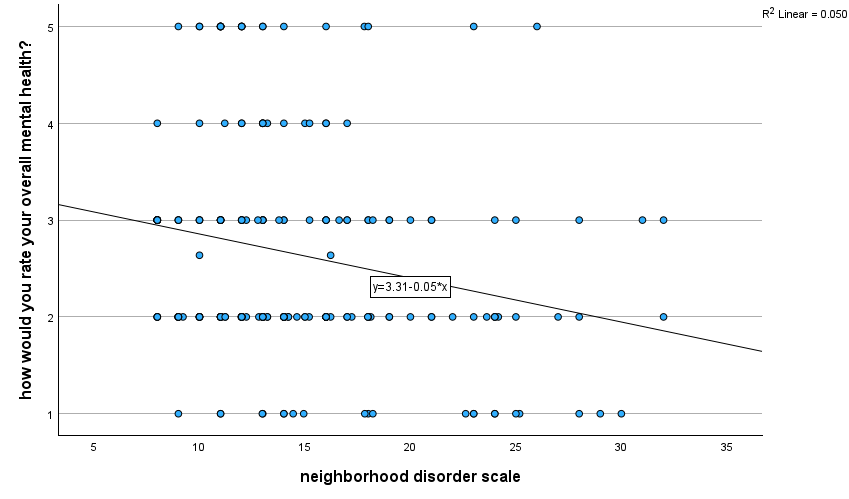 |
| **Neighborhood disorder and life satisfaction**  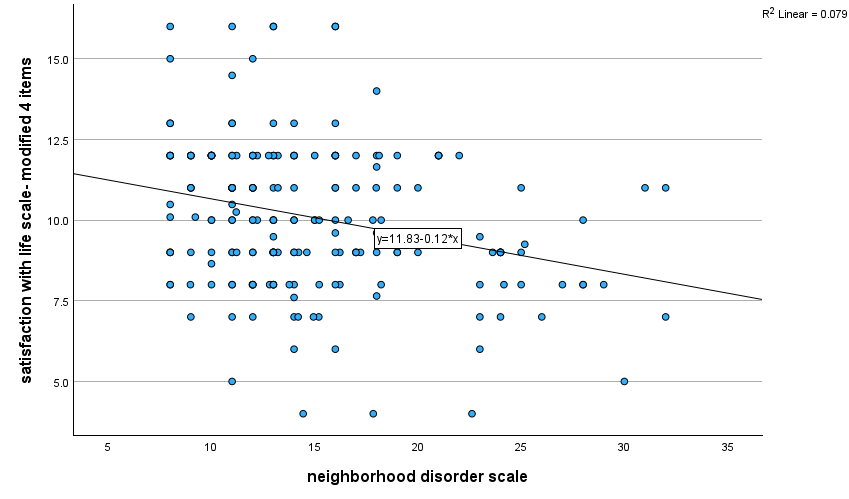 |
| **Neighborhood disorder and overall physical health**  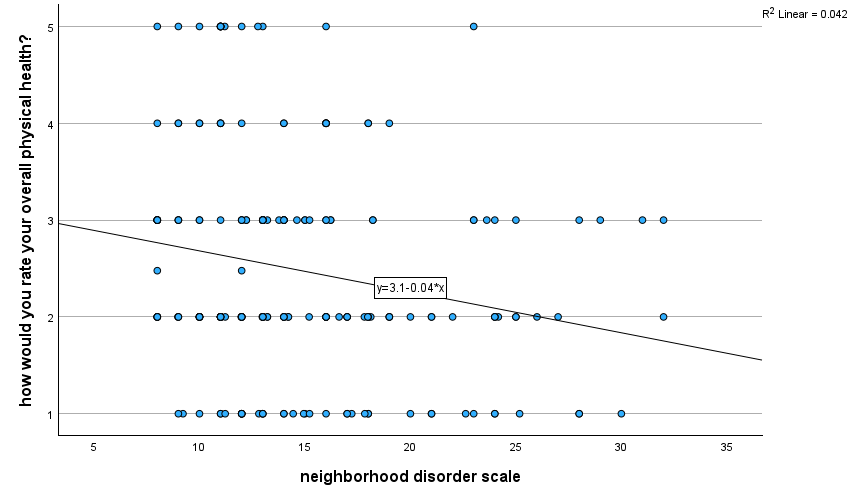 |
| **Neighborhood disorder and # of chronic health conditions**  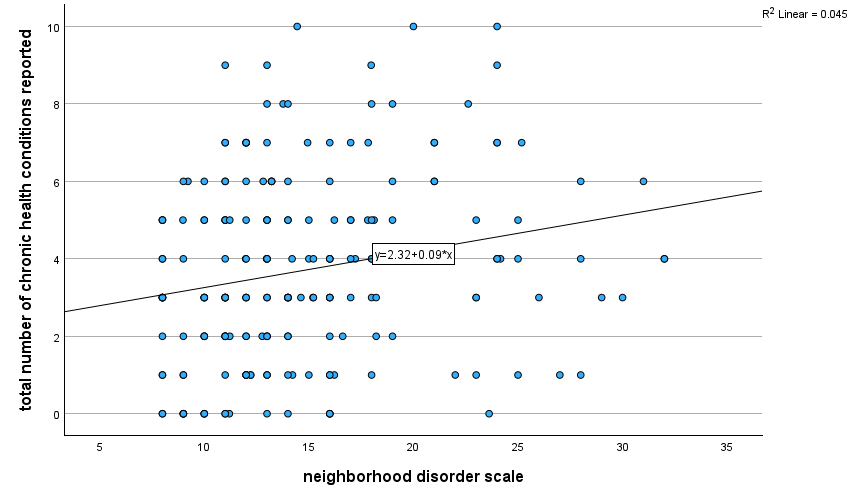 |
| **Neighborhood disorder and daily limitations** |
|  |


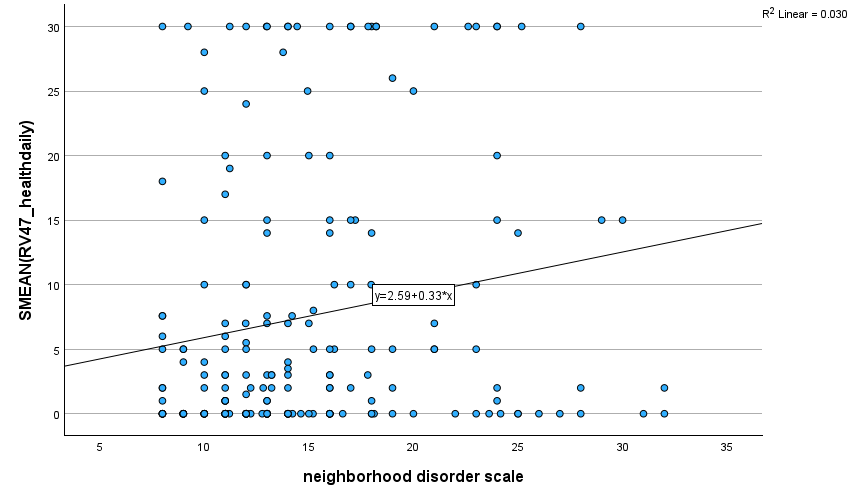


| **Normal Q Q plots**  **Food Insecurity**  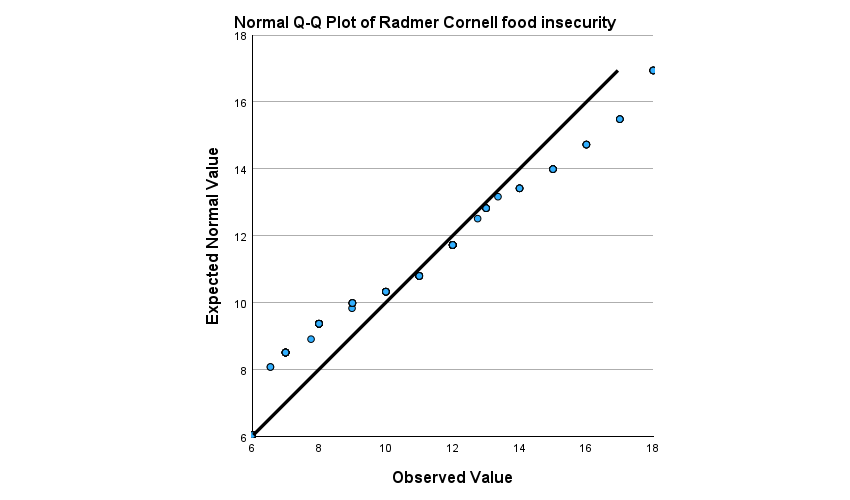  **Neighborhood disorder**  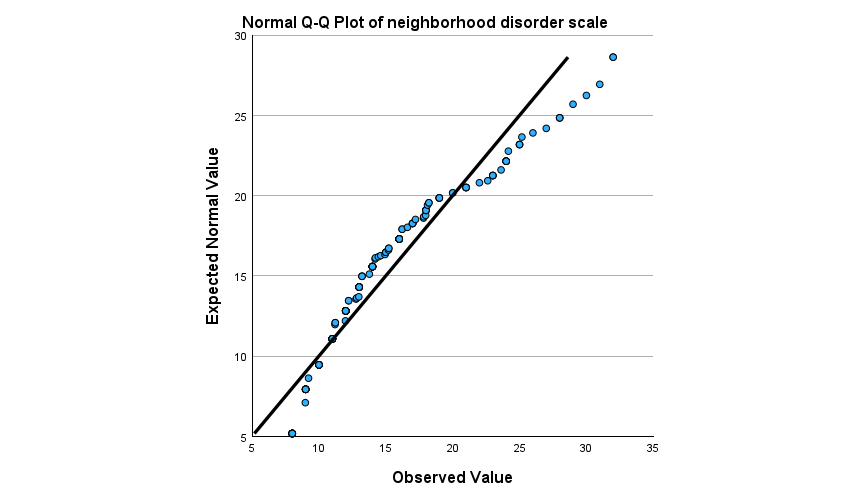  **Overall mental health**  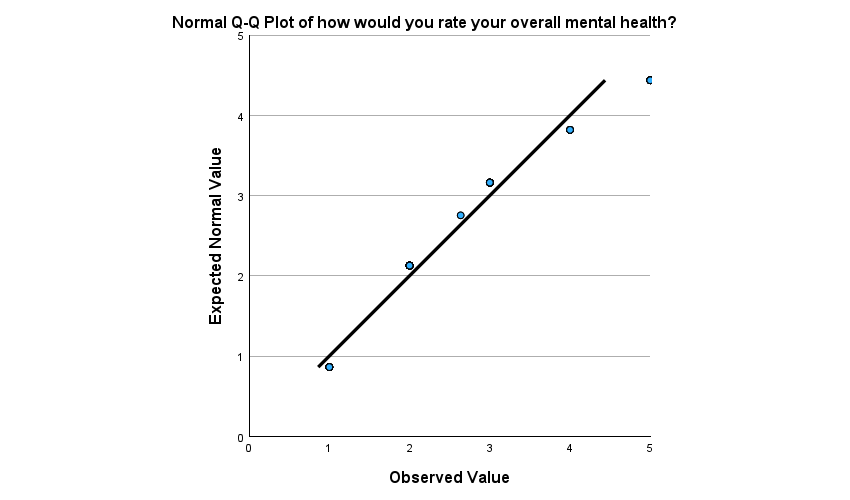  **Life satisfaction**  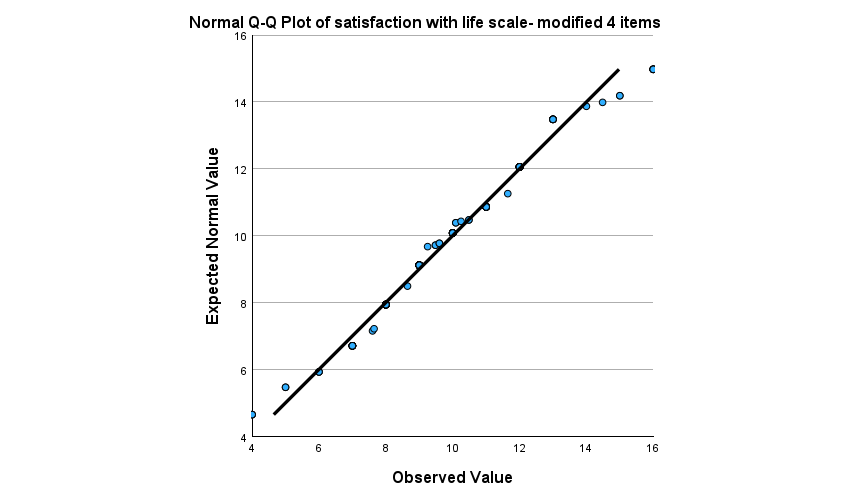  **Overall physical health**  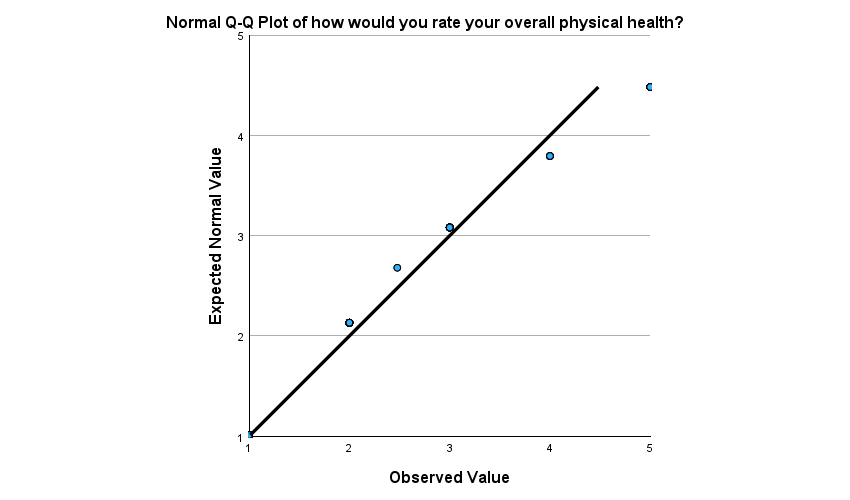  **Number of chronic health conditions**  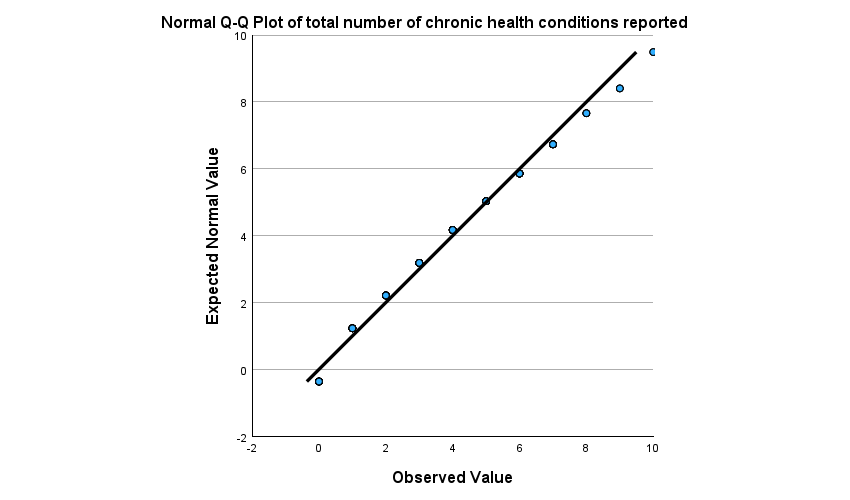  **Daily limitations** |
| --- |
| 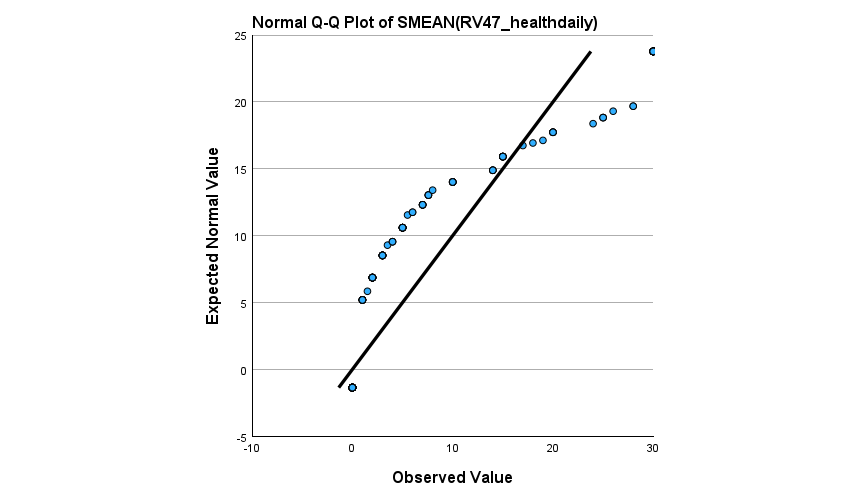 |
|  |
|  |
